# The X-linked inhibitor of apoptosis protein (XIAP) is up-regulated in metastatic melanoma, and XIAP cleavage by Phenoxodiol is associated with Carboplatin sensitization

**DOI:** 10.1186/1479-5876-5-6

**Published:** 2007-01-26

**Authors:** Harriet M Kluger, Mary M McCarthy, Ayesha B Alvero, Mario Sznol, Stephan Ariyan, Robert L Camp, David L Rimm, Gil Mor

**Affiliations:** 1 Department of Medicine, Yale University School of Medicine, 333 Cedar St, New Haven, CT 06520, USA; 2 Department of Obstetrics & Gynecology, Yale University School of Medicine, 333 Cedar St, New Haven, CT 06520, USA; 3 Department of Surgery, Yale University School of Medicine, 333 Cedar St, New Haven, CT 06520, USA; 4 Department of Pathology, Yale University School of Medicine, 333 Cedar St, New Haven, CT 06520, USA

## Abstract

XIAP up-regulation is associated with chemotherapy resistance. Phenoxodiol causes XIAP degradation and chemotherapy sensitization in ovarian cancer. Here we assessed XIAP expression in melanomas, using tissue microarrays containing 436 melanomas and 336 nevi by a novel method of automated, quantitative analysis (AQUA). We used S100 to define pixels as melanoma (tumor mask) within the array spot, and measured XIAP expression using Cy5-conjugated antibodies within the mask. XIAP expression was significantly higher in melanomas than nevi (P < 0.0001), and higher in metastatic than primary lesions (P < 0.0001). We then assessed a panel of melanoma cell lines for XIAP expression, and found high expression in all cell lines. Three of the cell lines were assessed for Phenoxodiol and Carboplatin sensitivity; all were resistant to Carboplatin and showed variable sensitivity to Phenoxodiol. Pre-treating Phenoxodiol sensitive cells with Phenoxodiol prior to Carboplatin resulted in XIAP degradation, associated with Carboplatin sensitization and apoptosis, whereas exposing Phenoxodiol resistant cells to Phenoxodiol resulted in less XIAP degradation and minimal Carboplatin sensitization. We conclude that XIAP levels in clinical specimens are significantly higher in melanomas than their benign counterparts, and higher in metastatic than in primary specimens, suggesting an association with malignant progression and disease aggression. Melanoma resistance to Carboplatin is possibly due to XIAP over-expression. Phenoxodiol can sensitize melanoma cells to Carboplatin *in vitro *with corresponding XIAP degradation, although the precise target and mechanism of action of Phenoxodiol are subject to further assessment. Targeting XIAP warrants additional investigation as a therapeutic approach for metastatic melanoma.

## Background

The incidence of cutaneous melanoma in the United States is rising faster than that of any other malignancy, with 62,190 expected new cases diagnosed in 2006 [1,2]. This increase in incidence, compounded by the lack of effective therapy once the disease has metastasized, underscores the need for improved methods of treating patients with unresectable melanoma. Melanoma is usually resistant to standard chemotherapy and radiation therapy. A number of chemotherapeutic and biological agents have activity in metastatic melanoma, albeit with disappointingly low response rates of less than 25% for any single agent or combination of agents, and none has improved overall survival when compared with observation [3-5]. Single agent dacarbazine remains one of the standard treatments, although the control arm of a recent clinical trial demonstrated a dismal response rate of only 6.8% to dacarbazine [6]. Single agent platinum and taxane drugs are viable alternatives, yielding comparable response rates of 3–20% [7,8]. Our understanding of mechanisms of resistance to chemotherapy is limited, as is our ability to overcome resistance, and new, well tolerated agents and approaches are required to sensitize melanoma cells to chemotherapy in order to improve outcome.

One of the most important mechanisms by which anti-cancer chemotherapy induces cell death is by activating the apoptotic cascade [9,10]. There are two chief apoptotic pathways, the death receptor pathway (the direct pathway) and the mitochondrial pathway (the indirect pathway). These pathways are described in detail in the literature [11]. The two pathways are inhibited by a group of proteins called the IAP (inhibitors of apoptosis) proteins, of which the X-linked inhibitor of apoptosis protein (XIAP) is a key member. XIAP (also known as hILP, MIHA and BIRC4) selectively binds and inhibits caspases -3, -7 and inhibits Apaf-1-cytochrome c-mediated actvitaion of caspase-9, but does not inhibit caspase-8, and thus is responsible for inhibiting much of the apoptotic pathways [12-14]. As opposed to other members of the IAP family, XIAP is the only member capable of direct inhibition of both the execution and initiation phases of the apoptotic cascade; it inhibits apoptotic activation by inhibiting caspase-9, and inhibits the execution phase by inhibiting the effector caspases, caspase-3 and caspase-7 [15]. The IAP proteins promote ubiquitination and proteasomal degradation of the caspases to which they bind via their RING-finger motif, which enables them to serve as E3 ubiquitin ligases [16]. XIAP has been shown in other cancers to be associated with resistance to chemotherapy [17-19], as well as resistance to radiation therapy [20,21]. Thus, targeting XIAP is likely to be a useful approach to overcoming resistance to chemotherapy. A number of preclinical studies have demonstrated efficacy of XIAP inhibitors for a variety of tumors [13,22,23]. Down-regulation of XIAP in melanoma cells by siRNAs sensitizes these cells to TRAIL-induced apoptosis [24].

Increased expression of XIAP has been shown to be associated with aggressive malignant behavior and disease progression in a number of malignancies, including lymphoma, breast cancer, lung cancer and renal cell carcinoma [18,25-27]. Zhang et al demonstrated up-regulation of XIAP in a limited number of melanoma cell lines [28], and Bowen et al demonstrated higher expression of XIAP in 4 melanoma cell lines than in normal melanocytes, and found an association between XIAP expression and resistance to chemotherapy and radiation therapy [29]. However, to the best of our knowledge, there are no large cohort studies that thoroughly assess expression levels of XIAP in specimens from patients with melanoma.

In previous studies we assessed the activity of Phenoxodiol, a novel isoflavone derivative, in ovarian cancer [30]. We showed that Phenoxodiol has activity as a single agent in patient-derived, chemotherapy-resistant ovarian cancer cell lines, via induction of Fas-mediated apoptosis. This effect is dependent upon the activation of the caspase system and inhibiting XIAP [30]. Subsequent studies showed that pretreatment of cells with Phenoxodiol prior to treatment with Docetaxel results in sensitization to Docetaxel, which is mediated by XIAP [17]. We showed that inhibition of XIAP by RNA interference yields sensitization to Docetaxel similar to that seen with Phenoxodiol [17]. Furthermore, we showed that pretreatment of chemotherapy-resistant ovarian cancer cells with Phenoxodiol results in decreases in XIAP levels and sensitization to Gemcitabine, Carboplatin and Paclitaxel [19]. Although the precise target of Phenoxodiol remains unknown, it inhibits XIAP by causing early, caspase independent self-ubiquitination and degradation of XIAP by the proteasome [19]. In these studies we showed that when cells are transfected with a phospho-mimic form of XIAP, which is resistant to proteasomal degradation, Phenoxodiol activity is inhibited, indicating that XIAP degradation is a critical component of Phenoxodiol-induced apoptosis.

Phenoxodiol has been assessed in Phase I and II studies, and is very well tolerated in both the oral and the intravenous forms [19,31]. The toxicities are limited to mild depression, lightheadedness, thrombocytopenia, hypertension and headache. Responses have been documented in platinum-resistant ovarian cancer, both as a single agent and when used in combination with platinum drugs [32].

Given that most melanoma patients do not respond to platinum-based therapy, we assessed the activity of Phenoxodiol in Carboplatin-resistant, early passage melanoma cell lines. We hypothesize that resistance of melanoma cells to chemotherapy is associated with high baseline expression of XIAP. We further hypothesize that drugs that (directly or indirectly) cause XIAP degradation can result in sensitization of melanoma cells to chemotherapy.

The purpose of the present work was to assess the expression patterns of XIAP in a large cohort of melanoma tumors and benign nevi, and to assess the association of expression with disease stage. In order to obtain quantitative, objective measures of expression, we used our newly developed method of automated, quantitative analysis (AQUA™) of tissue microarrays. This method has been validated, has proven to be more accurate than pathologist-based scoring of brown stain [33,34], and has been used in a number of prior melanoma studies [35-38]. AQUA provides continuous output scores that correspond to *in situ *XIAP levels in the melanocytes. We found significantly higher expression levels of XIAP in melanomas than in benign nevi. We also found global high expression in a panel of melanoma cell lines. We subsequently assessed the sensitivity of melanoma cell lines to Carboplatin, and found that all three cell lines studied were resistant to the drug. We assessed our ability to sensitize these cells to Carboplatin by pretreatment with Phenoxodiol, and the association with XIAP levels and XIAP cleavage.

## Materials and methods

### Tissue Microarray Construction

The melanoma tissue microarrays were constructed as previously described [35]. A total of 232 primary melanomas, 15 local recurrences and 299 metastatic cores, each measuring 0.6 mm in diameter, were spaced 0.8 mm apart on a glass slides. The cohort was constructed from paraffin-embedded, formalin-fixed tissue blocks obtained from the Yale University Department of Pathology Archives. Specimens and clinical information were collected under the guidelines and approval of a Yale University Institutional Review Board. The cohort has been used in prior publications [39-41]. The specimens were resected between 1959 and 2000, with a follow-up range between 2 months and 40 years, and a mean follow-up time of 6.7 years. Age at diagnosis ranged from 18 to 91 years (mean age 52.4 years). The cohort included 55% males and 45% females. The time between tumor resection and tissue fixation was not available. A pathologist reviewed slides from all of the blocks to select representative areas of invasive tumor to be cored. The cores were placed in the tissue microarray block using a Tissue Microarrayer (Beecher Instruments, Silver Spring, MD). The tissue microarrays were then cut into 0.5 *μ*m sections and placed on glass slides using an adhesive tape-transfer system (Instrumedics, Inc., Hackensack, NJ) with UV cross-linking. Similarly, a tissue microarray was made containing cores from 540 benign nevi. The nevus array contained 31 metastatic specimens from patients that were also represented on the melanoma array. Both arrays contained identical cell lines, cored from pellets, as previously described [42]. The overlapping metastatic specimens and cell lines were used for normalization of the scores obtained from the benign and malignant arrays.

### Immunohistochemistry

Staining was performed for automated analysis of melanoma specimens as previously described. Briefly, slides were deparaffinized in xylene, and transferred though two changes of 100% ethanol. For antigen retrieval, the slides were boiled in a pressure cooker containing 6.5 mM sodium citrate (pH 6.0). Endogenous peroxidase activity was blocked in a mixture of methanol and 2.5% hydrogen peroxide for thirty minutes at room temperature. To reduce non-specific background staining, slides were incubated at room temperature for 30 minutes in 0.3% bovine serum albumin/1× Tris-buffered saline. Slides were incubated at 4°C overnight in a humidity tray with a primary mouse anti-human XIAP antibody (BD Transduction Laboratories) at a dilution of 1:50. To create a tumor mask, slides were simultaneously incubated overnight with a primary rabbit anti-human S100 antibody diluted at 1:500. Slides were rinsed three times in 1× Tris-buffered saline/0.05% Tween-20 and incubated for 1 hour at room temperature with goat anti-mouse HRP to identify the target and goat anti-rabbit IgG conjugated to Alexa 546 to identify the S100 mask. The slides were washed again as above and incubated for ten minutes with Cy5 directly conjugated to tyramide (Perkin Elmer, Boston, MA) at a dilution of 1:50 for primary antibody identification. The slides were rinsed again and cover-slips were mounted with ProLong Gold antifade reagent, which contained 4,6-diamidine-2-phenylindole (DAPI) to identify the nuclei.

### Automated Image Acquisition

Images were acquired using our automated method, as described previously [33]. Briefly, areas of tumor were distinguished from stroma by creating a mask with the S100 signal tagged with Alexa 546. Coalescence of S100 at the cell surface was used to identify the membrane/cytoplasm compartment within the tumor mask, while 4,6-diamidino-2-phenylindole (DAPI) was used to identify the nuclear compartment within the tumor mask. The target marker, XIAP, was visualized with Cy5 (red). Cy5 was used because its emission peak is outside the color spectrum of tissue autoflourescence. Multiple monochromatic, high resolution (1024 × 1024 pixel 0.5-μm) grayscale images were obtained for each histospot, using the 10× objective of an Olympus AX-51 epifluorescence microscope (Olympus, Melville, NY) with an automated microscope stage. Digital image acquisition was driven by custom program and macro-based interfaces with IPLabs software (Scanalytics Inc., Fairfax, VA).

### Algorithmic Image Analysis

Images were analyzed using algorithms that have been previously extensively described [33]. Two images (one in-focus and one out-of-focus) were taken of the compartment specific tags and the target marker. A percentage of the out-of-focus image was subtracted from the in-focus image for each pixel, representing the signal to noise ratio of the image. An algorithm described as RESA (Rapid Exponential Subtraction Algorithm) was used to subtract the out-of-focus information in a uniform fashion for the entire microarray. Subsequently, the PLACE algorithm (Pixel Locale Assignment for Compartmentalization of Expression) was used to assign each pixel in the image to a specific subcellular compartment and the signal in each location was calculated. Pixels that could not accurately be assigned to a compartment were discarded. The data were saved and subsequently expressed as the average signal intensity per unit of compartment area. For the nuclear and membrane/cytoplasmic compartments, the image was measured on a scale of 0–255, and expressed as target signal intensity relative to the compartment area.

### Statistical Analysis

The JMP5 (SAS Institute Inc., Cary, NC) software package was used for data analyses. Continuous AQUA scores of target expression were divided by the median score and associations with clinical and pathological parameters were completed using the Chi-Square test. The prognostic significance of the parameters was assessed for predictive value using the Cox proportional hazards model with overall survival as an end point. Comparison of expression in malignant and benign specimens, as well as comparisons between primary and metastatic specimens was performed with unpaired t-tests.

### Cell Lines and Drugs

We used low passage (passage 3–18) melanoma cell lines established from tumors excised from patients treated at the Yale Cancer Center, obtained from the Cell Culture Facility of the Yale Skin Disease Research Core Center (YSDRCC). Cells were grown as previously described [43]. The melanoma cell line YUMAC was derived from an in-transit metastasis from a male patient, YUSAC2 was from a soft tissue metastasis from a male patient, and YUGEN8 was from a brain metastasis of a female patient. 501 mel, 624 mel and 888 mel were generously provided by Dr. Steven A. Rosenberg, National Cancer Institute, Bethesda, MD. The mm127 cell line was generously provided by Dr Peter Parsons of the Queensland Institute of Medical Research, Australia. Cells where grown in F12 medium with 10% FBS at 37°C in 5% CO_2_. Phenoxodiol was generously provided by Novogen, LTD, Australia. Carboplatin was purchased from Sigma Chemical Co. (St. Louis, MO).

### Cell Viability Assay

Cell viability was evaluated using the CellTiter 96 Aqueous One Solution Cell Proliferation Assay according to the manufacturer's instructions (Promega, USA), as previously described [44]. Briefly 4 × 10^4 ^cells per well were plated in triplicate in a 96-well microtiter plate (BD Biosciences). Cells were grown to 60% confluency, at which stage the medium was replaced with reduced serum phenol-depleted medium, OPTI-MEM (Gibco™, Invitrogen Corp, Grand Island, NY), and incubated for 4 hours prior to treatment. Following treatment, 20 μL of CellTiter 96 Aqueous One Solution was added to each well, and the plate was incubated at 37°C for 2 hours. Optical densities were measured at 490 nm using a Titertek Multiskan MCC/340 (Titertek Instruments Inc., Alabama). Values of treated cells were compared to untreated cells and reported as percent viability.

### Western Blot Analysis

Cells were plated in 35 mm^2 ^Petri dishes (BD Biosciences, San Jose, CA) and grown to 60% confluency for treatment. Following treatment, cells were lysed in Radioimmunoprecipitation (RIPA) buffer containing the protease inhibitor cocktail, Complete (Roche Diagnostics, Mannheim, Germany). Protein concentrations were calculated by the BCA (Bicinchoninic Acid) assay (Pierce Biotechnology, Rockford, IL). Protein (20 μg) was separated in a sample buffer [2.5% SDS, 10% glycerol, 5% β-mercapto-ethanol, 0.15 M Tris (pH = 6.8) and 0.01% bromophenol blue] and subjected to SDS-polyacrylamide gel electrophoresis using precast 12% polyacrylamide gels (Bio-Rad Laboratories, Hercules, CA), and transferred to pure nitrocellulose membranes (Bio-Rad). To inhibit non-specific binding, membranes were blocked in 5% powdered milk for 1 hour at room temperature. The membranes were washed three times in PBS with 0.5% tween (PBS-T) for 10 minutes per wash and incubated with primary antibodies diluted in PBS-T with 1% milk, in a 50 mL falcon tube on a rotator overnight at 4C. The following primary antibodies and concentrations were used: mouse anti-XIAP, (BD Transduction Laboratories) at 1:1000, mouse anti-Caspase-2 (BD Biosciences) at 1:1,000, rabbit anti-Bid (Cell Signaling, Beverly, MA) at 1:2500, and rabbit anti-actin (Sigma) at 1:100. Following primary incubation, membranes were washed as described above and incubated with horse anti-mouse or horse anti-rabbit peroxidase (Vector Laboratories, Burlingame, CA), diluted 1:10,000, for 1 hour at room temperature. Membranes were washed again in PBS-T as above and washed three times in ddH20, 10 minutes per wash. Finally, proteins were visualized using enhanced chemiluminescence (Pierce Biotechnology).

### Caspase -3, -8, and -9 activity

Following drug treatment of cells, 10 μg of protein in 50 μL of ddH_2_0 was combined with equilibrated Caspase-Glo™ 3/7, 8, or 9 reagents (Promega). After incubation for 1 hour at room temperature, luminescence was measured using a TD 20/20 Luminometer (Turner Designs, Sunnyvale, CA). Blank values were subtracted and fold-increase in activity was calculated based on activity measured from untreated cells. Each sample was measured in duplicate.

## Results

### XIAP expression in human tumors

**Figure 1:**
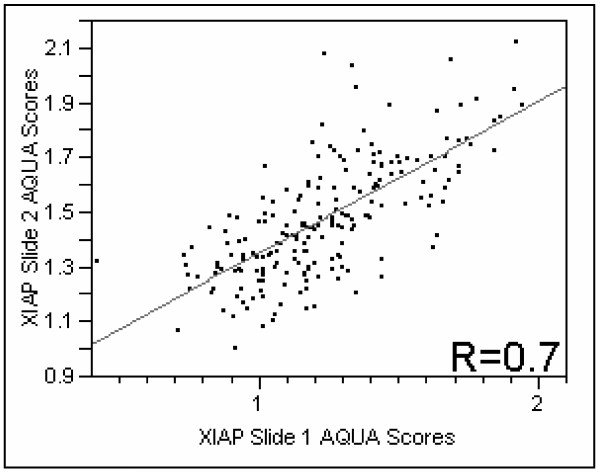
Regression plot of XIAP staining comparing scores from two slides containing histospots taken from the same patients, from different locations within the tumor.

To assess for intra-tumor heterogeneity of XIAP expression in melanoma specimens, we used tissue arrays containing tumor cores from 565 melanoma patients. Two separate slides, each containing a core from a different area of the tumor for each patient, were stained with a specific antibody for XIAP. Positive XIAP immunoreactivity was observed predominantly in the membranous/cytoplasmic compartment. The nuclear compartment had very low immunoreactivity, therefore only the membranous/cytoplasmic compartment was analyzed. We did not see differences in staining patterns within the tumor mask within a histospot. Using the Pearson correlation test, we found that the scores from matching spots on the two arrays were highly correlated (*P *< 0.0001) for XIAP expression. Figure 1 shows regression plots for the two arrays assessed for XIAP expression (R = 0.7).

**Figure 2:**
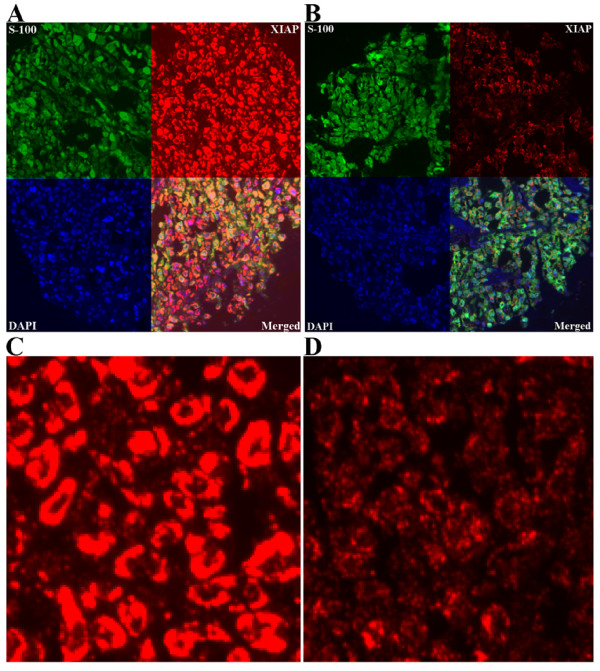
Strong (A) and weak (B) XIAP expression (red) in melanoma using ×10 magnification, employing S100 (green) to define tumor and DAPI (blue) to define nuclei. Strong (C) and weak (D) XIAP expression are shown using ×60 magnification. The strong spot corresponds to an AQUA score of 52.91 and the weak spot to a score of 21.85.

AQUA scores for melanoma specimens ranged from 6.07 to 109.91, with a median score of 23.47. Examples of strong and weak XIAP staining are shown in Figure 2. Figure 2C represents a histospot with an AQUA score of 52.91, and Figure 2D a score of 21.85.

AQUA scores from both slides were combined to give a single dataset. Of the 565 melanoma patients in the cohort, 228 had cores that were interpretable on both slides, and 283 were interpretable for one slide. Tumor spots were deemed uninterpretable if they had insufficient tumor cells, loss of tissue in the spot or an abundance of necrotic tissue. For patients who had two interpretable histo-spots, a composite score was formed by taking the average of the two scores. For patients with only one interpretable core, the single score was used. The combined dataset had scores for 436 melanoma patients; 195 primary specimens and 241 metastatic specimens. For the histospots on the nevus array, we obtained 336 scores.

**Figure 3:**
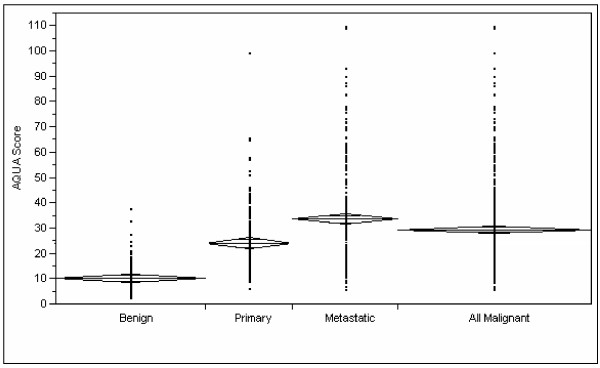
Unpaired t-tests showing significant differences in expression of XIAP between benign and malignant specimens (*p *< 0.0001), and significant differences between primary and metastatic lesions (*p *< 0.0001).

We assessed the differences in XIAP expression between benign and malignant tissue. Unpaired t-tests showed that expression was significantly higher in malignant versus benign tissue cores (*P *< 0.0001), as shown in Figure 3. Moreover, the expression of XIAP was significantly higher in metastatic specimens than in primary specimens (*P *< 0.0001), as shown in Figure 3. The mean AQUA scores for XIAP were 10.4 in nevi specimens, 24 in primary lesions and 33.7 in metastatic lesions.

AQUA gives continuous output data, as opposed to brown stain, which is read as "positive" or "negative". Here we define "low" or "normal" AQUA scores as those that fall at or below the 95^th ^percentile score for nevi. This score was 20 for XIAP, and scores above these cutoffs in the malignant specimens were considered "high". Table 1 demonstrates the association between high XIAP expression and commonly used clinical and pathological variables. High XIAP was associated with advanced stage (metastatic) disease and thick lesions over 2 mm (p = 0.0003 and p = 0.0264, respectively).

**Table 1:** Association between high XIAP expression and other prognostic clinical and pathological variables.

**Clinical/Pathological Variable**	**Chi-Square Value**	**P-value**
Disease Stage (metastatic vs. primary)	13.071	***0.0003***
Breslow (>2 mm)	4.927	***0.0264***
Clark Level (IV-V)	3.559	0.1687
Age (<40 years)	0.338	0.5613
Gender (male)	1.800	0.1798
Presence of Ulceration	2.016	0.3650

### XIAP expression in a panel of human cell lines

**Figure 4:**
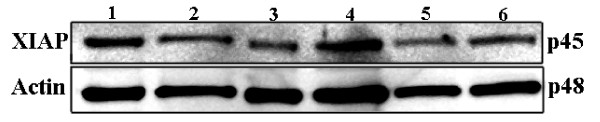
Expression of XIAP in melanoma cell lines. XIAP expression was determined by western blot analysis in 7 melanoma cell lines, ordered left to right: Mel 624 AB, Mel 624 MC, Mel 501, Mel 888, YUGEN8, YUMAC, MM127.

Given that chemotherapy response information was not available on most patients in our cohort, we sought to assess the association between high XIAP expression and chemotherapy resistance in melanoma using cell lines. We studied XIAP expression in a panel of melanoma cell lines by Western blot analysis. As shown in figure 4, all the cell lines studied expressed XIAP.

### Phenoxodiol inhibition of XIAP expression in melanoma cells

**Figure 5:**
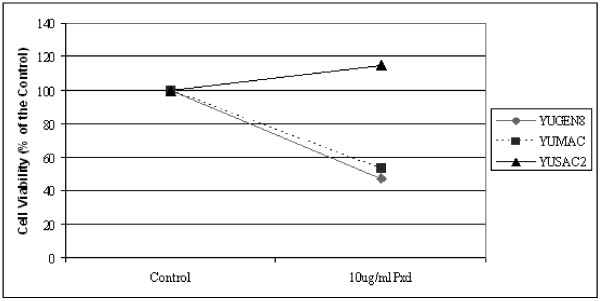
Cell viability assays demonstrating the effect of 10 μg/ml Phenoxodiol on three melanoma cell lines, YUMAC, YUSAC2 and YUGEN 8. Each experiment was performed in triplicate and viability was determined by the CellTiter 96 Aqueous One Solution Cell Proliferation Assay, reported as a percentage of viable cells relative to untreated cells.

A central effect of Phenoxodiol-induced apoptosis in different cell types, is degradation of XIAP [17,19]. Therefore we evaluated the effect of Phenoxodiol on three patient-derived melanoma cell lines. We used a concentration of 10 μg/ml, based on previous studies in ovarian cancer [19], and cell viability was determined by the CellTiter 96^® ^Aqueous One Solution Cell Proliferation Assay. As shown in Figure 5, two of the three cell lines (YUMAC and YUGEN8) were sensitive to 10 μg/ml of Phenoxodiol, while the third cell line (YUSAC2) was resistant. YUSAC2 cells were also resistant to Carboplatin (as detailed below) and surprisingly showed increased cell viability in the presence of Carboplatin compared to the control. This phenomenon has been demonstrated *in vivo *by other researchers who found that melanoma cell lines that were resistant to dacarbazine had higher growth and metastatic potential than untreated cells [45].

**Figure 6:**
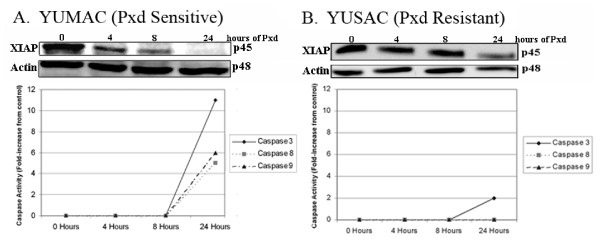
Effect of Phenoxodiol on YUMAC (A) and YUSAC2 (B) cells. Cells were treated with a single dose of Phenoxodiol (10 μg/mL) for varying time points (0, 4, 8, and 24 hours). XIAP and caspase-2 expression were determined by western blot analysis. Caspase-3, -8, and -9 activities were determined by the Caspase-Glo 3, 8, and 9 assays, respectively.

We then studied the effect of Phenoxodiol on XIAP expression and function in one of the two Phenoxodiol sensitive cell lines, YUMAC, and in the resistant cells (YUSAC2). Exposure of YUMAC cells to Phenoxodiol decreased the level of XIAP at 4 hours compared with pretreament levels. A further decrease in XIAP levels was seen at 8 hours post treatment and no expression was observed at 24 hours (Figure 6A). Changes in XIAP expression corresponded to increases in activity of caspases -3, -8 and -9 (Figure 6A). On the other hand, with the YUSAC2 cells, the degree XIAP degradation and resultant caspase activation was much less than what was observed with YUMAC cells (Figure 6B).

### Sensitization of melanoma cell lines to Carboplatin by Phenoxodiol and the association with XIAP levels

**Figure 7:**
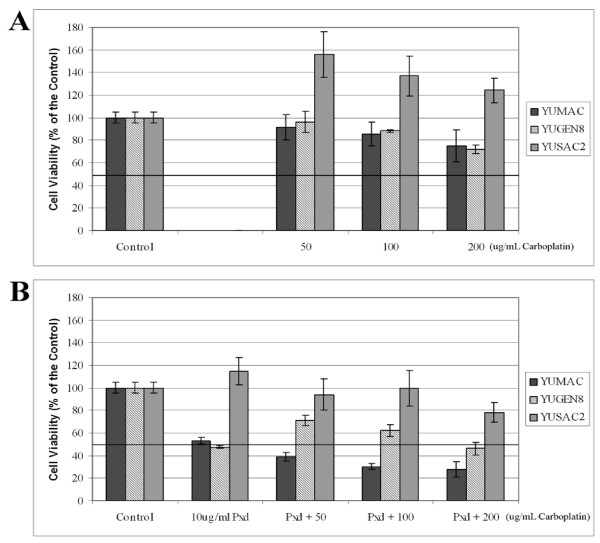
Cell viability assays demonstrating the effect of Phenoxodiol on melanoma cells. (A) Cells were treated with increasing concentrations of Carboplatin (50–200 μg/mL) alone or (B) cells were pretreated with 10 μg/mL Phenoxodiol for 4 hours followed by treatment with increasing concentrations of Carboplatin. Each experiment was performed in triplicate and viability was determined by the CellTiter 96 Aqueous One Solution Cell Proliferation Assay, reported as a percentage of viable cells relative to untreated cells.

YUMAC, YUSAC2 and YUGEN8 cells were evaluated for sensitivity to Carboplatin. The cells were treated with increasing concentrations of Carboplatin, ranging from 50–200 μg/ml for 24 hours, and cell viability was determined by the CellTiter 96^® ^Assay. As shown in Figure 7A, all three cell lines were resistant to Carboplatin, with YUSAC2 demonstrating the most resistance. As with Phenoxodiol, YUSAC2 cells grew more in the presence of Carboplatin than in medium alone. The IC_50 _for all three cell lines was greater than 200 μg/ml.

In previous studies we have shown that resistance of ovarian cancer cells to Carboplatin is associated with high XIAP levels, and that this resistance can be reversed by pretreatment with Phenoxodiol, which causes XIAP degradation and ubiquitination [19]. Here we assessed whether pretreatment of melanoma cells with Phenoxodiol reverses the baseline resistance to Carboplatin and whether this reversal is associated with XIAP degradation. Melanoma cells were pre-treated with 10 μg/ml of Phenoxodiol for 4 hours, Phenoxodiol was removed from the media and the cells were treated with increasing doses of Carboplatin (50–200 μg/ml) for an additional 24 hours. As shown in Figure 7B, pre-treatment for only 4 hours with Phenoxodiol sensitized the YUMAC and YUGEN8 cells to Carboplatin, as demonstrated by a decrease in the IC_50 _of Carboplatin for these cell lines to < 50 μg/ml and < 200 μg/ml, respectively. Pretreatment with Phenoxodiol for 4 hours did not significantly reduce the resistance of YUSAC2 to Carboplatin, with the IC_50 _for Carboplatin remaining at > 200 μg/ml.

**Figure 8:**
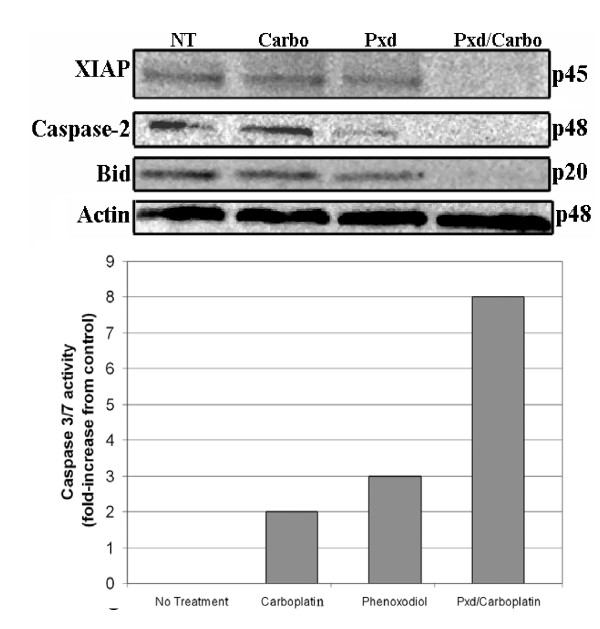
Effect of pretreatment with Phenoxodiol on the apoptotic cascade. YUMAC cells received either no treatment, 200 μg/mL of Carboplatin for 24 hours, 10 μg/mL of Phenoxodiol for 2 hours, or 10 μg/mL of Phenoxodiol for 2 hours followed by 200 μg/mL Carboplatin for 24 hours. Both pro- and antiapoptotic protein expression was determined by Western blot analysis. Activity of caspase-3 was determined using the Caspase-Glo 3\7 assay.

In order to study the intracellular effect of Phenoxodiol on melanoma cells, we pretreated YUMAC cells with a shorter exposure to Phenoxodiol (2 hours) followed by treatment with Carboplatin. We assessed the effect on some apoptotic mediators, including pro-caspase-2, BID (a member of the mitochondrial pathway that is activated after caspase-2 activation) XIAP and caspase-3/7. Treatment with Carboplatin alone had no effect on XIAP levels, or the other components of the apoptotic cascade. Treatment with Phenoxodiol for 2 hours had some effect on XIAP expression and induced caspase-2 activation. When Carboplatin was added after Phenoxodiol pre-treatment, remarkable XIAP degradation was observed, coupled with caspase-2 and Bid activation (as demonstrated by disappearance of the bands representing the inactive forms of caspase-2 and Bid), and a significant increase on the activity of caspase-3/7 (Figure 8).

## Discussion

The purpose of this study was to 1) assess the expression of XIAP in a quantitative fashion on a large cohort of melanoma specimens in an objective, automated fashion, and to compare expression of XIAP in malignant specimens to benign nevi and 2) assess the association between XIAP expression and resistance to Carboplatin, and the effect of Phenoxodiol on XIAP. In our large cohort of melanomas and nevi we show significantly higher expression in tumors than in nevi, and XIAP expression was significantly higher in metastatic specimens than in primary melanomas. Among primary tumors we found an association between high XIAP expression and deep lesions (>2 mm). Furthermore, we demonstrate that degradation of XIAP by Phenoxodiol confers Carboplatin sensitization in melanoma cells.

One of the major problems in the treatment of unresectable melanoma is the inherent resistance to chemotherapy; response rates to any single chemotherapeutic agent or combination of agents are in the order of 25% at best, and the responses are typically not durable [46]. Chemotherapy resistance in melanoma cells is multifactorial. Mechanisms implicated in chemoresistance in melanoma include (but are not limited to) multi-drug resistance transporter proteins [47,48], oxidative agents such as nitric oxide [49], DNA repair pathways [50] and intrinsic resistance to apoptosis, as reviewed by Soengas et al [51].

Chemotherapy resistance associated with defects in the apoptotic cascade is due to the over-expression of factors that inhibit the apoptotic signal initiated by cytotoxic agents. XIAP is one of the major inhibitors of apoptosis, due to its ability to block both the intrinsic and extrinsic pathways. We did not have information on chemotherapy treatment and response to therapy on the patients included in this cohort. We are currently collecting specimens from a randomized clinical trial in which metastatic melanoma patients on the control arm are being treated with chemotherapy alone, and we will assess XIAP levels and the association with chemotherapy response.

A number of drugs that directly target XIAP are in development, including antisense oligonucleotide molecules (AEG 35156, Aegra pharmaceuticals, Montreal, Canada) and small molecule inhibitors, as reviewed by Schimmer et al [15], and these inhibitors might enhance chemosensitivity in XIAP over-expressing tumors. Finding significantly higher expression in melanoma than in nevi was encouraging for development of XIAP-targeting therapies in melanoma. However, there was some overlap in the range of expression between benign nevi and melanoma specimens, particularly for primary melanomas. It is unclear why some nevi had relatively high XIAP levels; although these nevi were not thought to be "atypical" on pathologic evaluation, some benign melanocytes might be undergoing early malignant transformation. Given that these benign nevi were excised, we cannot assess the association between XIAP expression and clinical outcome in nevi. Moreover, other researchers have shown that somatic mutations are already evident in nevi, but not in normal skin, such as the presence of activating B-raf and N-ras mutations [52,53]. Because level of expression may be an important determinant of sensitivity to XIAP targeted therapies, we sought to determine the percent of samples that displayed XIAP over-expression as compared to nevi. For this purpose, we defined high expression as exceeding the AQUA score in 95% of benign nevi, as done in prior studies [41]. We found that 61% of the primary and 77% of the metastatic specimens met our definition for high expression. While it is unknown whether there is a threshold level above which XIAP inhibitors might be more effective as therapeutics, our data show that there is variability in XIAP expression in melanoma, which should, at the very least, be assessed when XIAP targeting therapies are used in clinical trials in melanoma patients.

Extensive research has been done on the structure and function of XIAP, as reviewed in detail by Schimmer et al [15]. In addition to caspase inhibition, XIAP has a role in activation of the JNK pathway, with resultant MAP kinase pathway activation, leading to NF-κB activation [54]. XIAP also activates NF-κB by promoting its nuclear translocation [55]. XIAP mediates cell cycle arrest, via regulation of cyclins and cyclin-dependent kinase inhibitors (CDKIs), and is involved in receptor-mediated signaling [56].

Targeting XIAP to sensitize cancer cells to chemotherapy is supported by a number of studies showing that over-expression of XIAP in cell lines confers chemotherapy resistance, and that down-regulation of XIAP results in activation of both the mitochondrial and death receptor pathways and sensitization to chemotherapy [17,19,57-59]. Targeting XIAP is unlikely to result in significant toxicity as XIAP knock-out mice exhibit normal organ function [60]. Although clinical toxicity data using the XIAP targeting drug AEG 35156 are pending, we believe that the approach of targeting XIAP in order to sensitize melanoma cells to chemotherapy is worthy of further investigation.

Phenoxodiol is an isoflavone derivative that has been shown to cause proteasomal degradation of XIAP, and reverse chemoresistance in ovarian cancer cells [19]. The precise target and mechanism of action of Phenoxodiol remain unknown, and XIAP degradation might be a downstream effect of Phenoxodiol, rather than a direct effect of the drug. In ovarian cancer we showed that both knock down of XIAP by RNA interference and pretreatment with Phenoxodiol result in similar sensitization to chemotherapy [17]. In this study we demonstrated that Phenoxodiol treatment of melanoma cells also caused XIAP degradation, cleavage of pro-caspase-2, and activation of caspases -3, -8 and -9 in Carboplatin resistant melanoma cell lines. The pro-apoptotic effect of Phenoxodiol on melanoma cells potentiated the cytotoxic effects of Carboplatin, and reduced the IC_50 _of Carboplatin. We showed that in our Carboplatin resistant cells, caspase-3 (the main effector caspase) was not activated by Carboplatin alone, yet pre-treatment of YUMAC cells with Phenoxodiol for 2 or 4 hours resulted in decreased levels of XIAP, sensitization to Carboplatin, and remarkable activation of caspase-3. We note that in the YUSAC2 cells there was much less XIAP degradation and no meaningful sensitization to Carboplatin. These results confirm previous studies by our group and others suggesting that intracellular inhibitors of caspases, such as XIAP, play a major role in drug-induced apoptosis [17,19,58], and by triggering caspase activation, Phenoxodiol enhances the cytotoxic effect of chemotherapy. Although the molecular differences between these two cell lines have yet to be determined, these results elucidate potential new targets for chemoresistance. We are presently screening additional potential target(s) mediating this resistance.

Single agent Carboplatin is not commonly used for melanoma, whereas Cisplatin is widely used. The chemical structure of these drugs is very similar, and in most (but not all) diseases these drugs have demonstrated equal efficacy. Carboplatin has been used as a singe agent in Phase II studies for metastatic melanoma, with response rates of 11–16% [61,62]. Use of single agent Cisplatin in Phase II trials has resulted in response rates of 10–16% [7,63]. While these two platinum compounds have not been compared head-to-head for melanoma, the toxicity associated with Cisplatin often prohibits its use in patients, and further development of sensitizers to Carboplatin is a logical approach. Phenoxodiol has been assessed in Phase I studies, and is extremely well tolerated in both the oral and the intravenous forms [19,31,64]. In our *in vitro *studies we used a concentration of 10 μg/ml of Phenoxodiol in order to sensitize cells to Carboplatin. In clinical trials with oral Phenoxodiol given at a dose of 50 mg/kg, the serum concentration was in the order of 250 μM after 30 minutes of administration [19]. Thus the dose used in this study (10 μg/ml = 41.62 μM) is readily attainable *in vivo*, and is likely to be well tolerated. Of the three cell lines we assessed for sensitization to Carboplatin, one remained resistant after Phenoxodiol pre-treatment. Studies are underway in our laboratory to assess additional isoflavone analogues as chemotherapy sensitizers, using a larger panel of cell lines. However, in select patients whose tumors demonstrate XIAP degradation with Phenoxodiol treatment, use of Phenoxodiol as a chemotherapy sensitizer is a reasonable approach that warrants further investigation. These clinical trials should incorporate assessment of XIAP degradation in short term cultures after treatment with Phenoxodiol as a biomarker of response to this combination of therapy. This is currently being assessed in platinum resistant ovarian cancer patients treated with Phenoxodiol and Cisplatin.

In summary, our studies show that XIAP is up-regulated in melanoma specimens compared to nevi, and expression of XIAP is higher in metastatic than in primary melanomas. This is the first study of XIAP expression in a large cohort of melanoma specimens. Furthermore, we demonstrate that Phenoxodiol sensitization to Carboplatin in melanoma is associated with XIAP degradation, although the precise target of Phenoxodiol is still being studied in our laboratory. Further studies are needed to determine whether isoflavone analogues and direct XIAP targeting therapies can be used as chemotherapy sensitizers in melanoma patients, and whether XIAP expression levels are associated with response to therapy.
